# Association between Salivary Cortisol Levels, Dental Anxiety, and Dental Caries in Children: A Cross-Sectional Study

**DOI:** 10.3390/dj11090205

**Published:** 2023-08-29

**Authors:** Vivek Padmanabhan, Md Sofiqul Islam, Muneera Habib, Zainab Abdulaziz, Manjunatha Goud, Nallan CSK Chaitanya, Sheela Haridas, Muhammed Mustahsen Rahman

**Affiliations:** 1 RAK College of Dental Sciences, RAK Medical and Health Sciences University, Al Juwais, Al Qusaidat, Ras al Khaimah 11172, United Arab Emirates; sofiq.islam@rakmhsu.ac.ae (M.S.I.);; 2 RAK College of Medical Sciences, RAK Medical and Health Sciences University, Al Juwais, Al Qusaidat, Ras al Khaimah 11172, United Arab Emiratessheela.hardias@rakmhsu.ac.ae (S.H.)

**Keywords:** dental caries, dental anxiety, salivary biomarker, stress, cortisol, children

## Abstract

Aim: This study aimed to investigate the relationship between dental caries, dental anxiety, and salivary cortisol levels in children visiting pediatric dental clinics and their implications on pediatric oral health. Materials and Methods: A cross-sectional study was conducted at a dental university in the UAE. A total of 60 children, aged 4–12 years, were included. Salivary cortisol levels were measured using an Enzyme-Linked Immunosorbent Assay (ELISA) kit. Dental caries status was evaluated, and dental anxiety levels were assessed using the Modified Dental Anxiety Scale (MDAS). Statistical analyses, including Mann-Whitney U test and Pearson’s correlation coefficient, were performed to determine significant differences and associations. Results: The study group showed significantly higher salivary cortisol levels compared to the control group (*p* < 0.0001). A strong positive correlation was found between salivary cortisol levels and dental caries status (*p* < 0.001). However, no significant difference in dental anxiety levels was observed between the study and control groups (*p* = 0.85). A strong positive correlation was found between dental anxiety levels and dental caries status (*p* < 0.001). Conclusion: The findings indicate a significant association between salivary cortisol levels and dental caries, suggesting that higher cortisol levels are associated with active caries. Dental anxiety levels were positively correlated with dental caries. Understanding the relationship between these variables can contribute to better oral health strategies and interventions for children, emphasizing the importance of managing dental anxiety and stress in pediatric dental care. Clinical Significance: This study highlights the potential of salivary cortisol as a biomarker for assessing stress and its impact on oral health in children. By addressing dental anxiety and stress, dental professionals can provide child-friendly dental care, enhance preventive measures, and improve oral health outcomes in pediatric patients.

## 1. Introduction

Dental caries, or tooth decay, is a common oral health issue affecting children worldwide. It is characterized by the destruction of tooth structure due to bacterial activity and dietary factors [1]. Dental caries in children can lead to pain, difficulty in eating, and other complications [2]. Preventive measures, such as proper oral hygiene practices and regular dental check-ups, are crucial in minimizing the impact of dental caries and promoting optimal oral health in children [1,2,3]. Dental anxiety is a prevalent issue among children when visiting the dentist. It involves fear, apprehension, and stress related to dental procedures. Dental anxiety in children can lead to avoidance of dental care, compromising their oral health [4]. It is important to address dental anxiety through child-friendly dental practices, effective communication, and behavioral management techniques [5,6]. By creating a positive and supportive environment, dental professionals can help alleviate dental anxiety and ensure optimal oral health outcomes in children [4,6]. Saliva is increasingly recognized as a valuable diagnostic tool in healthcare. Its non-invasive collection and rich composition make it ideal for assessing biomarkers related to various conditions [7]. Salivary diagnostics offer potential for early detection, monitoring disease progression, and personalized treatment approaches, revolutionizing existing healthcare practices [7]. Cortisol, commonly known as the stress hormone, is a vital hormone released by the adrenal glands in response to stress [8]. It plays a crucial role in regulating the body’s stress response, influencing various physiological processes. Cortisol levels fluctuate throughout the day, but prolonged or excessive stress can lead to chronically elevated levels, which can have detrimental effects on physical and mental health [8,9]. Salivary cortisol levels in children have garnered interest as a potential indicator of stress and its impact on health [9]. Cortisol, a hormone released in response to stress, can be non-invasively measured in saliva. Understanding salivary cortisol levels in children is valuable for assessing their physiological stress response, evaluating the influence of stress on various health outcomes, and exploring the association between cortisol, dental caries, and dental anxiety [10]. Investigating salivary cortisol levels provides insight into the intricate relationship between stress and children’s oral health [9,11,12].

This study was designed to understand the relationship between dental caries, dental anxiety and salivary cortisol levels in children visiting the pediatric clinics of a dental university in UAE. The null hypothesis of this study was that there is no significant association between salivary cortisol levels, dental anxiety and dental caries in children.

## 2. Materials and Methods

This was a cross sectional study conducted at RAK College of Dental Sciences (RAKCODS), RAK Medical and Health Sciences University (RAKMHSU), RAK, UAE. The objective was to assess the association between dental caries, dental anxiety, and salivary cortisol levels by evaluating the salivary cortisol levels in children with and without dental caries, as well as evaluating the dental anxiety levels in children visiting dentists. This research was approved by the Research and Ethics committee of the university and the RAK Research and Ethics Committee, Ministry of Health, (Proposal/Approval number: RAKMHSU-REC-036-2022/23-UG-D, RAK-REC-REF-12-2023-UG-D). Children who visited RAKCODS pediatric clinics for their dental treatment were recruited after obtaining consent from the parents and assent from the children. The consent from parents and the assent from children were recorded. The children recruited for the study were between the ages of 4–12 years. Children whose parents did not provide consent or had any medical condition were not included in the study so as to prevent any bias in the findings of the study. The basic demographic data was recorded and then the oral hygiene evaluation sheet used for the community services of the institution was used to evaluate the oral health and record the findings including DMFT/dmft status of the child. The children were divided into study and control groups based on the presence or absence of dental caries. Thirty children were included in the study group with at least five active carious lesions. The study group consisted of thirty children, each having a minimum of five active carious lesions. The authors deliberately selected children with five active carious lesions to ensure a structured and homogeneous sample, thereby minimizing the possibility of chance occurrences in the study’s findings. Thirty children with no dental carious lesions were included into the prospective control group. The children enrolled in the study typically visited the clinics between 3:30 p.m. to 6:30 p.m., corresponding to the regular operating hours of the pediatric dentistry clinic. After obtaining consent and assent from both the children and their parents, appointments were scheduled. Prior to their visit, the participants were instructed to refrain from eating or drinking anything for a minimum of 2 h. Additionally, they were asked to rinse their mouths before saliva collection to ensure a standardized procedure. The principal investigator provided comprehensive training to all co-investigators regarding data recording and dental examination procedures for children. To prevent any potential bias in recordings, one co-investigator was responsible for data recording during clinic visits, while another was trained to conduct intraoral examinations. Additionally, one co-investigator received specialized training in saliva sample collection to avoid examiner bias. For salivary cortisol estimation, unstimulated saliva samples were collected after obtaining consent from both guardians and children, where participants were instructed to slightly bend their head forward, relax, and passively drool accumulated saliva for 5 min into a graduated tube using the Coachman’s Position; the saliva samples were then stored at 4 °C in an icebox and promptly sent to a laboratory within 20 min for examination of cortisol levels using an Enzyme-Linked Immunosorbent Assay (ELISA) kit [13]. Children who were not co-operative were not included in the study. The Modified Dental Anxiety Scale (MDAS) [12]., was utilized to assess the dental anxiety levels of the children in the study. The MDAS is a well-established and commonly used tool designed to measure dental anxiety in both research and clinical settings. It consists of a questionnaire that includes several statements related to dental anxiety, and participants rate their agreement or disagreement with each statement using a numerical scale. The individual item scores are then summed to obtain a total score, which provides a quantitative measure of the participant’s overall level of dental anxiety. By employing the MDAS, the researchers aimed to obtain reliable and standardized data on the children’s dental anxiety, enabling a comprehensive analysis of its impact on the study’s outcomes. A pre-structured questionnaire based on socio-demographic details and oral hygiene habits was used for data collection. The dental examination was done before the children were subjected to dental treatment within the clinics. The Principal investigator had trained all the co-investigators as to the method of recording the data and also the dental examination of children. The oral hygiene sheet, the MDAS scale, and the collection of saliva was done by one of the co-investigators only so as to prevent any collection bias.

The data were analyzed by the statistical program SPSS version 29 (IBM Corp. Released 2022. IBM SPSS Statistics for Windows, Version 29.0. Armonk, NY: IBM Corp). The purpose of the analysis was to compare the mean Salivary Cortisol Levels between two groups: the study group and the control group. The comparison was done using the Mann-Whitney U test, which is a non-parametric test used to determine if there is a significant difference between the distributions of two independent groups. Additionally, the analysis aimed to assess the correlations between Salivary Cortisol Levels, dental anxiety, and dental caries. Pearson’s correlation coefficient was used for this purpose. Pearson’s correlation coefficient measures the strength and direction of the linear relationship between two continuous variables. In this case, it was used to determine the degree of association between Salivary Cortisol Levels, dental anxiety, and dental caries. To determine statistical significance, a *p*-value threshold of less than 0.05 was used. By conducting these statistical analyses, the authors aimed to gain insights into the potential differences in Salivary Cortisol Levels between the study and control groups, as well as the relationships between Salivary Cortisol Levels, dental anxiety, and dental caries. These findings can help in understanding the impact of these variables on dental health and inform future research or interventions in this area.

## 3. Results

**Figure 1:**
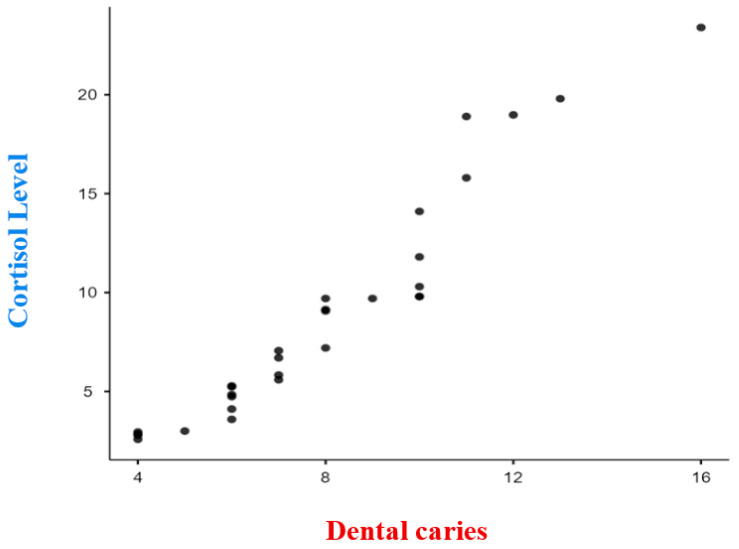
Correlation between dental caries and Salivary cortisol level. As dental caries increases; the Salivary Cortisol Levels also increase. There is a strong positive correlation (0.9597) which is statistically significant (*p* ≤ 0.0001).

**Figure 2:**
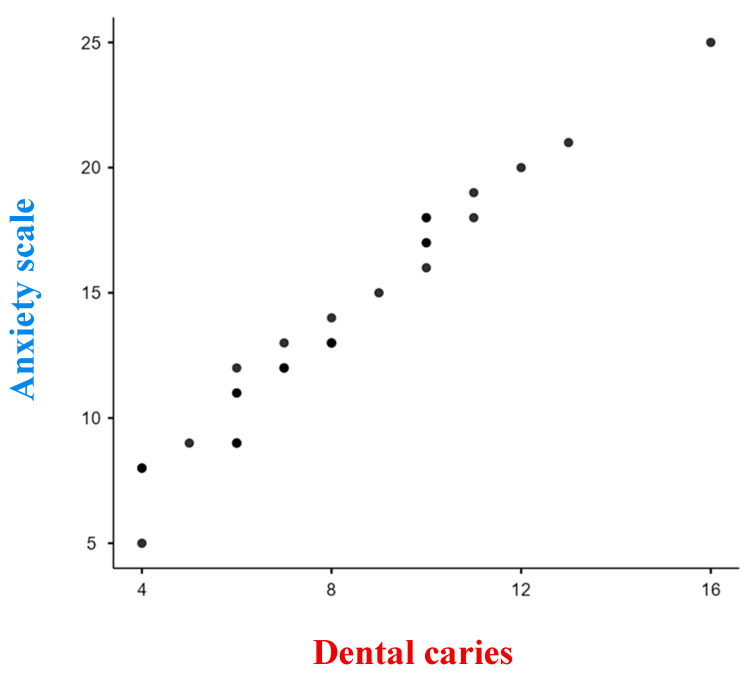
Correlation between dental caries and dental anxiety score. As dental caries increases; there is an increase in dental anxiety levels. There is a strong positive correlation (0.9803) which is statistically significant (*p* ≤ 0.0001).

In this study, the researchers aimed to assess a group of 60 children who visited dental clinics for treatment. The sample included an equal number of boys and girls, with 30 females and 30 males. The age range of the children was from 4 to 12 years, with a mean age of 7.45 ± 1.35 years. The study collected data on three main variables: dental caries status (dmft/DMFT), dental anxiety using the MDAS scale [12]., and salivary cortisol levels measured using an ELISA kit. Table 1 in the study presents the comparison of mean salivary cortisol levels between children with active caries (study group) and those without any active cavities (control group). The mean salivary cortisol levels in the study group were found to be 5.8213 ng/mL, while the mean levels in the control group were 1.133 ng/mL. The reported *p*-value of less than 0.0001 (*p* < 0.0001) indicates that this difference in salivary cortisol levels between the two groups is statistically significant. Furthermore, the study analyzed the correlation between salivary cortisol levels and dental caries status (dmft/DMFT) within the study group using Pearson’s correlation coefficient. In this case, it was used to assess the association between salivary cortisol levels and dental caries. The reported correlation coefficient of 0.9597 and a *p*-value of less than 0.0001 indicate a strong positive correlation between salivary cortisol levels and dental caries, which is statistically significant (Figure 1). In Table 2, the study presents the comparison of dental anxiety levels between the study group and the control group. The study group exhibited a mean dental anxiety level of 13.5, whereas the control group showed a mean of 13.73. The obtained *p*-value of 0.85 indicates that the observed difference in dental anxiety levels between the two groups lacks statistical significance. Additionally, the study examined the correlation between dental anxiety levels and dental caries status (dmft/DMFT) within the study group using Pearson’s correlation coefficient. The reported correlation coefficient of 0.9803 and a *p*-value of less than 0.0001 indicate a strong positive correlation between dental anxiety levels and dental caries, which is statistically significant (Figure 2). Overall, the findings of the study suggest a statistically significant association between salivary cortisol levels and dental caries, indicating that higher salivary cortisol levels are associated with active caries. Similarly, a statistically significant association was found between dental anxiety levels and dental caries, indicating that higher dental anxiety levels are associated with the presence of caries. However, no statistically significant difference in dental anxiety levels was observed between the study and control groups. Top of Form.

## 4. Discussion

The primary aim of this research was to assess the interplay among dental caries, dental anxiety, and salivary cortisol levels in the context of pediatric dental treatment. By examining these key variables, the study sought to gain valuable insights into their complex interactions, which could inform the development of more effective oral health strategies and interventions for children. Understanding the relationship between these factors has the potential to enhance preventive measures and promote better oral health outcomes in pediatric dental care. The study focused on three key variables: dental caries status, dental anxiety, and salivary cortisol levels. Understanding the factors that contribute to the development of dental caries and the impact of dental anxiety on oral health is essential for providing effective dental care to children. Moreover, salivary cortisol levels, which serves as an indicator of stress, has been suggested to have implications on oral health outcomes. Therefore, this study aimed to examine the relationship between dental caries, dental anxiety, and salivary cortisol levels in the context of pediatric dental treatment. By investigating these variables, the study aimed to gain valuable insights into the complex interactions among dental caries, dental anxiety, and stress markers. Such insights could contribute to the development of improved oral health strategies and interventions for children. Understanding the relationship between these factors has the potential to enhance preventive measures and promote better oral health outcomes in pediatric dental care settings.

When the salivary cortisol levels were compared in children with and without dental caries, the salivary cortisol levels were found to be increased in children with dental caries when compared to the salivary cortisol levels in children without dental caries and the results were statistically significant (Table 1). When individuals are exposed to stressful situations, the hypothalamic-pituitary-adrenal (HPA) system releases neurotransmitters and hormones that elicit fear and dampen brain activity [14]. Cortisol, in particular, plays a crucial role in the activation of the HPA axis following stress. The response to stress varies among individuals based on factors such as personality, physical strength, and overall health. Psychosocial variables like stress, depression, and anxiety have been strongly linked to periodontal diseases, and chronic stress can impact oral health in various ways. Stress can lead to a decrease in salivary flow rate, and corticosteroids can induce atrophic changes in major salivary glands, altering saliva composition and volume. Although no study has directly examined the relationship between salivary cortisol secretion and dental properties, exposure to therapeutic corticosteroids has been shown to cause hypoplasia, which increases susceptibility to dental caries [15]. Salivary cortisol, despite accounting for 50–60% of plasma cortisol levels, can serve as a valuable marker for assessing adrenocortical function and stress levels [14,15,16]. Previous research has explored the use of salivary cortisol as a biomarker for stress, with some studies focusing on its connection to early childhood caries (ECC) [10]. However, the relationship between salivary cortisol levels and dental caries experience in children remains unclear. While some studies have not found significant differences between children with and without caries, others have reported that dental treatment reduced salivary cortisol levels in children with caries, but these levels remained higher compared to caries-free children [11]. Furthermore, studies have demonstrated significantly higher salivary cortisol levels in children with ECC compared to those without caries [10]. In the present study the salivary cortisol levels was found to have a positive correlation with increased experience of dental caries (dmft/DMFT status) in children and the results were statistically significant (Figure 1). The findings were similar to other studies which evaluated similar variables [14,17,18,19,20]. The increase in salivary cortisol levels with increased dental caries experience can be attributed to several factors. Firstly, dental caries is often associated with pain and discomfort, which can trigger a stress response in individuals. Stress activates the hypothalamic-pituitary-adrenal (HPA) axis, leading to the release of cortisol [14]. Secondly, chronic inflammation and infection associated with dental caries can contribute to systemic inflammation and dysregulation of the HPA axis, resulting in elevated cortisol levels [19]. Additionally, the psychological impact of dental caries, such as anxiety and fear related to dental visits and treatment, can also contribute to increased cortisol production [20].

The present study aimed to explore the association between dental anxiety and dental caries in children. The findings revealed a significant relationship between increased dental anxiety and a higher prevalence of dental caries among the participants. This aligns with previous research indicating that higher levels of dental anxiety are often observed in individuals with a history of dental caries [20]. The link between dental anxiety and dental caries can be explained through several mechanisms. Firstly, children with higher dental anxiety may exhibit avoidance behaviors, leading to delayed or infrequent dental visits, and subsequently inadequate oral hygiene practices. Poor oral hygiene can contribute to the accumulation of dental plaque, which is a primary factor in the development of dental caries [21]. Secondly, dental anxiety may lead to increased physiological stress responses during dental visits. Elevated stress levels can affect salivary flow and composition, potentially reducing the protective effects of saliva against dental caries [19]. Stress-induced changes in saliva can affect the buffering capacity and alter the balance of oral microorganisms, promoting the growth of cariogenic bacteria and increasing the risk of dental caries [21]. Furthermore, dental anxiety can also influence oral health-related behaviors, such as dietary choices. Individuals with high dental anxiety may be more prone to consuming cariogenic foods and beverages, contributing to the development and progression of dental caries [21,22,23]. In the present study it was found that there was a strong positive correlation between dental caries experience and dental anxiety levels suggesting that with increased dental anxiety the dmft/DMFT or dental caries status increased. Studies have shown a positive correlation between dental caries and dental anxiety in children, indicating that higher levels of dental anxiety are associated with an increased risk of dental caries [14]. Dental anxiety refers to the fear, apprehension, and stress experienced by individuals when visiting the dentist or undergoing dental procedures [14]. The relationship between dental caries and dental anxiety can be explained by several factors. Firstly, children with higher levels of dental anxiety may avoid or delay dental visits, leading to inadequate oral hygiene practices and an increased susceptibility to dental caries. Dental anxiety can act as a barrier to seeking regular dental care and receiving timely preventive interventions, thereby contributing to the development and progression of dental caries [24]. Secondly, dental anxiety can impact oral health behaviors. Children with dental anxiety may exhibit poor oral hygiene practices, such as infrequent or inadequate tooth-brushing, and may avoid necessary dental treatments. These behaviors can further increase the risk of dental caries [25]. Additionally, the physiological and psychological responses associated with dental anxiety can influence the development of dental caries. Increased levels of stress and anxiety during dental procedures can lead to elevated salivary cortisol levels, which can disrupt saliva composition and flow [24]. Changes in saliva, including reduced flow and altered pH levels, can create an environment conducive to the growth of cariogenic bacteria and the development of dental caries [25]. Furthermore, the negative emotional experiences associated with dental anxiety, such as pain, discomfort, and perceived lack of control, may contribute to a cycle of avoidance and negative reinforcement [24]. Children who associate dental visits with negative experiences may develop heightened anxiety and exhibit avoidance behaviors, further increasing their vulnerability to dental caries [24,25].

This study offers significant findings regarding the connection between dental caries and salivary cortisol levels in children. Nevertheless, it is crucial to acknowledge certain limitations that warrant consideration. The cross-sectional design employed in this study restricts our ability to establish a causal relationship between dental caries and salivary cortisol levels. To gain a deeper understanding of the temporal dynamics between stress, salivary cortisol, and dental caries, longitudinal studies are necessary. Furthermore, while salivary cortisol levels were used as an indicator of stress in this study, it is important to recognize that other stress markers and psychological factors might also contribute to the relationship and should be explored in future research. The chronic stress that the present study refers to in children with long standing dental caries can be validated using specialized questionnaires to understand the presence of chronic stress. Moreover, the validation of chronic stress can be further strengthened by conducting hair cortisol analysis, which serves as an additional indicator of long-term stress presence. Although chronic stress can impact physiological processes and lead to increased cortisol levels, the evidence supporting a direct causative link between stress and dental caries is limited. In this study, cortisol levels were elevated, while anxiety levels remained mostly unchanged, implying that the heightened salivary cortisol levels may be temporary and possibly triggered by the stress associated with dental visits. The study’s findings suggest that dental caries alone might not strongly influence anxiety levels in children. However, the observed variations in cortisol levels indicate a significant physiological response to the anticipation of dental treatment or a visit to the dentist. This acute reaction to the dental setting implies that the increased cortisol levels are more likely a short-term stress response rather than a result of chronic, long-term stress. Also the authors feel that future studies can be improvised by the use of social and behavioral questionnaire which can assess the presence of factors like peer pressure, bullying or any other factors which can elevate stress or anxiety levels in children and if these have any impact on oral health. By addressing these limitations, future investigations can provide a more comprehensive understanding of the complex interplay between stress, salivary factors, and dental caries in children. 

In conclusion, this study provides valuable insights into the association between dental caries and salivary cortisol levels in children, further supporting the notion that stress-induced hormonal changes, as indicated by elevated salivary cortisol, may contribute to an increased risk of dental caries. Understanding the impact of stress and salivary factors on oral health outcomes is crucial for developing preventive strategies and interventions targeting stress management to promote better oral health in children. Further research in this area will enhance our understanding of the complex relationship between stress and dental caries, leading to improved preventive and therapeutic approaches in pediatric dentistry. Furthermore, the positive correlation between dental caries and dental anxiety in children highlights the importance of addressing both the psychological and oral health needs of pediatric patients. Implementing strategies to reduce dental anxiety, such as utilizing behavior guidance techniques, effective communication skills, and creating a child-friendly dental environment, can help alleviate anxiety, improve dental experiences, and ultimately reduce the risk of dental caries in children. Additionally, early intervention, education, and promoting positive oral health behaviors are crucial in mitigating the impact of dental anxiety on oral health outcomes in children. By integrating psychological approaches and tailored dental care, dental professionals can optimize oral health outcomes and enhance the overall well-being of pediatric patients. 

## 5. Recommendations for Dentists and Pediatric Dentists Treating Pediatric Patients:

Salivary Cortisol as a Biomarker: The study highlights salivary cortisol’s potential as a stress-assessment biomarker in children’s oral health. Pediatric dentists can consider incorporating it for identifying stress-related oral health issues.

Managing Dental Anxiety: The strong correlation between dental anxiety levels and dental caries emphasizes the importance of addressing dental anxiety in pediatric care. Creating child-friendly environments, using behavior management techniques, and providing support can help alleviate anxiety during dental visits.

Preventive Measures: Understanding the relationship between dental caries, dental anxiety, and salivary cortisol can improve oral health strategies for children. Doctors should emphasize preventive measures like regular check-ups, oral hygiene practices, and dietary counseling.

Enhancing Oral Health Outcomes: Addressing dental anxiety and stress in pediatric dental care can lead to better oral health outcomes. Holistic approaches, including psychological support, ensure overall well-being in young patients.

## 6. Conclusions

In conclusion, this study highlights the significant association between dental caries, salivary cortisol levels, and dental anxiety in children. The findings suggest that stress-induced hormonal changes, as reflected in elevated salivary cortisol, may contribute to an increased risk of dental caries. Recognizing the impact of stress and addressing dental anxiety are crucial for developing preventive strategies and interventions to promote better oral health outcomes in children. By integrating psychological approaches and tailored dental care, dental professionals can effectively reduce dental anxiety and improve oral health in pediatric patients.

## Figures and Tables

**Table 1:** Comparison of Mean Salivary Cortisol Levels between study and control group.

Group	Mean	SD	*p* Value
Study	8.8213	5.71	<0.0001
Control	1.133	1.1648	

The mean Salivary cortisol levels of Control group (1.133 ng/mL) are significantly less than those of Study group (8.8213 ng/mL) and the results are statistically significant (*p* value < 0.0001).

**Table 2:** Comparison of dental anxiety score in children with and without dental caries.

Group	Mean	SD	*p* Value
Study	13.5	4.63	0.858
Control	13.73	5.42	

The results of mean dental anxiety score of Study group (13.5) and Control group (13.7) are not statistically significant (*p* value = 0.85842).

## Data Availability

All data are available upon request.
